# A-Kinase Anchoring Protein-Lbc: A Molecular Scaffold Involved in Cardiac Protection

**DOI:** 10.3390/jcdd5010012

**Published:** 2018-02-08

**Authors:** Dario Diviani, Halima Osman, Erica Reggi

**Affiliations:** Département de Pharmacologie et de Toxicologie, Faculté de Biologie et de Médecine, Lausanne 1005, Switzerland; Halima.Osman@unil.ch (H.O.); Erica.Reggi@unil.ch (E.R.)

**Keywords:** A-kinase anchoring protein (AKAP), protein kinase A, cyclic AMP, cardiomyocyte, cardiac protection, signal transduction

## Abstract

Heart failure is a lethal disease that can develop after myocardial infarction, hypertension, or anticancer therapy. In the damaged heart, loss of function is mainly due to cardiomyocyte death and associated cardiac remodeling and fibrosis. In this context, A-kinase anchoring proteins (AKAPs) constitute a family of scaffolding proteins that facilitate the spatiotemporal activation of the cyclic adenosine monophosphate (AMP)-dependent protein kinase (PKA) and other transduction enzymes involved in cardiac remodeling. AKAP-Lbc, a cardiac enriched anchoring protein, has been shown to act as a key coordinator of the activity of signaling pathways involved in cardiac protection and remodeling. This review will summarize and discuss recent advances highlighting the role of the AKAP-Lbc signalosome in orchestrating adaptive responses in the stressed heart.

## 1. Introduction

The heart responds to various stresses and insults such as increased blood pressure, myocardial infarction, and exposure to drugs and toxicants by undergoing a remodeling process that leads to heart failure, a lethal condition in which the cardiac output cannot satisfy the oxygen needs of the body [1,2,3]. Cardiac remodeling can be associated with an initial adaptive phase where ventricular cardiomyocytes undergo compensatory hypertrophy to maintain cardiac function [4,5]. However, in the long term, hypertrophy predisposes to adverse ventricular events associated with cardiomyocyte death, fibrosis, and progressive cardiac dysfunction [3,6,7]. Heart failure has an annual incidence of 1% in the population over 65 and a five-year survival rate after diagnosis lower than 50% [4]. This underscores the urgent need of identifying new therapies for this syndrome. In this respect, defining key protective signaling pathways favoring survival of cardiomyocyte subjected to stress could provide new opportunities to prevent cardiac remodeling and dysfunction under pathophysiological situations associated with heart injury or insults. 

A-kinase anchoring proteins (AKAPs) are molecular scaffolds that act as signal organizers. They ensure coordination of multiple signaling pathways at discrete microdomains of cardiomyocytes and cardiac fibroblasts by locally recruiting the cAMP-dependent protein kinase (PKA) as well as other signaling enzymes [8,9,10,11]. Anchoring of PKA is mediated by conserved domains constituted by amphipathic helices of about 20 amino acids [12,13], whereas targeting of AKAP-based signaling complexes to distinct subcellular sites is achieved through specialized protein- or lipid-binding domains located on the anchoring proteins [10]. Among the multitude of signaling molecules recruited by AKAPs one can find kinases, phosphodiesterases (PDEs), adenylyl cyclases (ACs), phosphatases, and GTPases [14,15,16,17,18,19,20]. In this respect, the assembly of signaling enzymes displaying opposing action (i.e., kinases and phosphatases) allows bidirectional regulation of transduction events, whereas the clustering of activators and downstream targets (i.e., ACs and PKA substrates) promotes signal potentiation [18]. 

So far, about 17 AKAPs have been identified in cardiac tissues [21,22,23] and shown to regulate various homeostatic, adaptive as well as pathophysiological functions including heart rhythm and action potential propagation, calcium cycling and cardiac contraction, cardiac remodeling and heart failure, as well as cardiac protection [5,24,25,26]. This suggests that modulating the ability of AKAP complexes to locally coordinate the activity of signaling molecules might have major impact on the function of the stressed and/or diseased heart and could be exploited to promote protection and maintain cardiac function. In particular, AKAP-Lbc (AKAP13), a heart-enriched anchoring protein [17], has been shown to organize diverse signaling pathways favoring protection against a cardiac stresses including pressure overload, as well as drugs and toxicants [27,28,29]. The current minireview article will focus on the role of this multifunctional anchoring protein in favoring adaptive and survival responses in the injured heart. In recent years, additional AKAPs have been show to confer cardiomyocyte protection either in vitro or in vivo including D-AKAP-1 (AKAP1), AKAP79/150 (AKAP5) mAKAP (AKAP6) and AKAP12. For more information about the cardioprotective role of these AKAP-based signaling complexes we refer the reader to other recent publications [30,31,32,33,34,35,36].

## 2. The Role of the cAMP/PKA Pathways in Cardiac Protection

Studies undertaken during the last 20 years indicate that activation of the cAMP/PKA signaling pathway can protect cardiomyocytes against cell death and damage induced by ischemia/reperfusion, anthracycline treatment, hyperglycemic stress, and pressure overload. Early experiments performed using isolated rats hearts demonstrated that cardiac cAMP levels and PKA activity are increased during ischemic preconditioning and that suppression of cAMP signaling attenuates myocardial protection against sustained ischemia [37,38]. Several additional studies later showed that preconditioning of mouse, rat or rabbit hearts with various Gs-coupled receptor agonists including isoproterenol (ISO) [39], glucagon-like peptide 1 (GLP-1) [40], adrenomedullin [41], corticotropin releasing factor [42], and adiponectin [43], confers protection against subsequent ischemia and reduces infarct size in a PKA-dependent manner. Similarly, PKA has also been shown to mediate the protective effects of the GLP-1 receptor agonist exendin-4 against hyperglycemia-induced cardiomyocyte apoptosis [44], of the antidiabetic drug metformin against anthracycline cardiotoxicity [45], and of adrenomedullin 2 against pressure-overload induced cardiac remodeling [46]. 

These protective effects rely on the ability of PKA to regulate multiple effector proteins and responses in cardiomyocytes. On the one hand, protection against ischemia/reperfusion has been shown to rely on the ability of PKA to (1) inhibit calpain-dependent proteolysis and degradation of structural proteins in cardiomyocytes [38]; (2) increase the opening of the mitochondrial Ca^2+^-activated K^+^ (mitoK(Ca)) channels and improve the efficiency of mitochondrial energy production [41]; (3) promote phosphorylation and enhance the cardioprotective effects of the small heat-shock protein HSP20 [47,48]; (4) reduce inhibitor of Kappa B (IκB) phosphorylation and nuclear factor Kappa B (NF-κB) activation [43]; (5) reduce nicotinamide adenine dinucleotide phosphate (NADPH) oxidase overexpression and superoxide overproduction [43]; and (6) improve calcium handling through phospholamban (PLB) phosphorylation and sarcoplasmic reticulum Ca^2+^ ATPase 2 (SERCA2) activation [49].

On the other hand, protection against pathological cardiac remodeling requires PKA mediated-regulation of histone deacetylases (HDACs) 4 and 5 [50,51]. These two signaling molecules control the activity of transcription factors, such as the myocyte enhancer factor 2 (MEF2), crucially involved in the regulation of gene programs associated with cardiac remodeling [52]. PKA induces HDAC4 proteolysis and the formation of an N-terminal HDAC cleavage product that inhibits the activity of MEF2 [50]. Moreover, the kinase also phosphorylates HDAC5, which, in turn, prevents its nuclear export, leading to the inhibition of MEF2-dependent transcription and fetal gene expression [51]. However, since these later findings where obtained using primary cultures of cardiomyocytes as a model system, investigation should be pursued to determine whether regulation of HDAC function by PKA has anti-remodeling effects in hearts subjected to various forms of stress. 

Interestingly, PKA reduces detrimental cardiac remodeling not only by protecting cardiomyocytes from dysfunction and death but also by inhibiting cardiac fibrosis. In this respect, it has been recently shown that activation of PKA signaling by prostaglandin E_2_ receptor 4 (EP4) agonists significantly prevented progression of myocardial fibrosis in response to pressure overload [53]. Experiments performed using isolated cardiac fibroblasts subsequently indicated that PKA activation suppresses collagen overproduction induced by the profibrotic agonist transforming growth factor β1 (TGF-β1) [53]. This suggests that PKA might attenuate the formation interstitial cardiac fibrosis, and consequent heart dysfunction through the reduction of excessive extracellular matrix deposition. 

The studies described above were carried out using activators or inhibitors that impact cardiac PKA signaling in a global manner and do not allow the precise identification of specific PKA functions in the heart. To circumvent this problem, several studies now adopt more targeted approaches and investigate the function of individual AKAP-PKA signaling complexes in specific cardiac cellular populations. 

## 3. AKAP-Lbc Signaling and Cardiac Protection

AKAP-Lbc (AKAP13) is a cardiac enriched anchoring protein [17], which functions as a scaffold for multiple signaling enzymes as well as a guanine nucleotide exchange factor (GEF) that selectively activates the small molecular weight GTPases RhoA and RhoC [17,23,54]. The exchange of GDP for GTP and the binding to Rho-GTPases is ensured by tandem Dbl-homology (DH) and plekstrin-homology (PH) domains located in the middle of the anchoring protein [17,54,55]. This central catalytic core is surrounded by N-terminal and C-terminal sequences, which provide anchoring sites for signaling molecules [23], and inhibit the basal Rho-GEF activity of AKAP-Lbc in the absence of stimulatory signals [17]. Deletion of these key regulatory regions, which has been shown to occur in chronic myeloid leukemia (CML) patients as the consequence of a chromosomal translocation between chromosomes 15 and 7, significantly increases the basal Rho-GEF activity and promotes oncogenic transformation [55,56]. 

The Rho-GEF activity of AKAP-Lbc is enhanced by G-protein-coupled receptors (GPCRs) linked to the heterotrimeric G protein G12 such as α1-adrenergic receptors (α1-ARs) [57]. In this respect, it has been shown that the α subunit of G12 (Gα12) can directly activate AKAP-Lbc by binding to a docking site located in its C-terminus. This interaction is proposed to suppress autoinhibitory intramolecular bonds between C-terminal regulatory sequences and the GEF region of the anchoring protein [58].

Initial in vitro studies performed in primary cultures of rat neonatal cardiomyocytes (NVMs) indicated that AKAP-Lbc acts as a mediator of the hypertrophic effects induced by α1-AR and endothelin 1 receptor (ET1-R) agonists [57,59]. These findings served as base for subsequent in vivo investigations showing that the anchoring protein mediates early adaptive growth responses that allow the heart to functionally compensate biomechanical or neurohumoral stresses [27,28]. Finally, in recent years, it became evident that AKAP-Lbc also coordinates and regulates signaling molecules such as the mitogen activated protein kinase (MAPK) p38α [27,60], protein kinase D1 (PKD1) [28,59], and the heat shock protein 20 (HSP20) [61], that promote adaptive and/or cytoprotective responses in cardiomyocytes. The following sections will discuss how coordination of distinct signaling pathways by the AKAP-Lbc signaling complex contributes to cardiomyocyte adaptation and protection against to various stressors and toxicants. 

### 3.1. AKAP-Lbc Mediates Protection against Pressure Overload-Induced Cardiac Dysfunction

Left ventricular pressure overload can be triggered by chronically elevated systemic blood pressure or obstructions of the outflow tract such as aortic valve stenosis. It initially leads to cardiac hypertrophy, which eventually may become maladaptive and predispose to heart failure. It is estimated that chronic hypertension doubles the risk of developing heart failure [4]. Experimentally, pressure overload can be induced in the mouse by transverse aortic constriction (TAC). Cardiac AKAP-Lbc is significantly upregulated in mice subjected to TAC as well as in patients with hypertrophic cardiomyopathy [27,28,59]. It assembles a macromolecular signaling complex coordinating the activity of transduction enzymes such as p38α and PKD1 that have a direct impact on compensatory hypertrophy and maintenance of cardiac function during the early phase of cardiac remodeling. 

#### 3.1.1. The Role of AKAP-Lbc-Mediated Regulation of p38α

**Figure 1:**
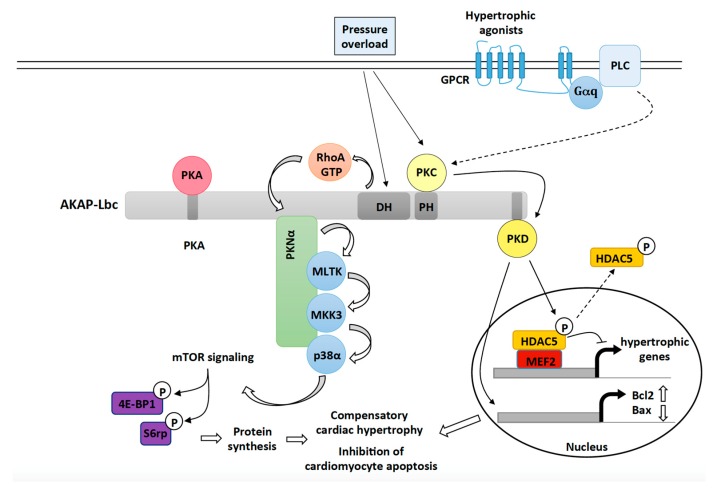
The role of the AKAP-Lbc signaling complex in mediating compensatory cardiac hypertrophy and cardiac protection in response to hemodynamic and neurohumoral stresses. Upon pressure overload, AKAP-Lbc promotes the formation of RhoA-GTP, which, in turn, triggers a signaling cascade involving anchored PKNα, MLTK, MKK3 and p38α. Activated p38α, through an unknown mechanism, enhances mTOR activity resulting in increased phosphorylation of 4E-BP1 and ribosomal protein S6 (S6rp), which leads to enhanced protein synthesis and cardiomyocyte growth. Pressure overload as well as activation of Gq-coupled receptors by hypertrophic agonists (ET-1, Angiotensin II) also promote the activation of AKAP-Lbc-anchored PKD1, which, in turn, phosphorylates HDAC5 and favors its nuclear export. As a result, MEF2 becomes activated and promotes transcription of hypertrophic genes. Activated PKD1 plays protective roles during compensatory hypertrophy by inducing the expression of antiapoptotic genes such as Bcl-2 and by inhibiting transcription of pro-apoptotic genes such as Bax.

The role of p38α in cardiac adaptation to stress has been subject of discussion over the last decade. Initial investigations suggested that chronic (constitutive) activation or inhibition of cardiac p38α does not affect hypertrophy [62,63,64,65,66]. However, subsequent studies overturned this view by showing that inducible activation of p38α signaling in adult hearts promotes cardiomyocyte growth [67,68]. In cardiomyocytes, AKAP-Lbc forms a p38-activating transduction unit that includes p38α and its upstream activators protein kinase N α (PKNα), mixed lineage kinase-like mitogen-activated protein triple kinase (MLTK), and mitogen-activated protein kinase kinase 3 (MKK3) (Figure 1) [27]. Cardiomyocyte-specific overexpression of a molecular disruptor of the interaction between AKAP-Lbc and PKNα inhibits pressure overload-induced p38α activation and compensatory cardiac hypertrophy. This leads to the appearance of early signs of heart failure including left ventricular dilation, increased cardiomyocyte apoptosis, and depressed cardiac function [27]. The ability of the AKAP-Lbc/p38α complex to promote compensatory hypertrophy is linked to the induction of mammalian target of rapamycin (mTOR) and the consequent increase in protein synthesis (Figure 1) [27]. These results indicate that AKAP-Lbc facilitates activation of p38α and mTOR in response to abrupt increases in the afterload to promote hypertrophy and reduce cell death, which temporarily preserves the function of the stressed heart. While the pathway linking the AKAP-Lbc/p38α complex and mTOR is currently unknown, recent findings indicate that p38 can enhance cardioprotective mTOR signaling by regulating the activity of the tuberous sclerosis complex (TSC) [69]. 

#### 3.1.2. The Role of AKAP-Lbc-Mediated Regulation of PKD1

Early work by Carnegie et al. showed that AKAP-Lbc can interact with PKD1 and PKCη (Figure 1) [70]. They could demonstrate that stimulation of rat NVMs with agonists binding Gq-coupled receptors, such as α1-ARs and ET1-Rs, enhances PKC activity, which, in turn, phosphorylates anchored PKD1 at serine 944 and 948 to induce its activation. PKD1 is released from the complex when PKA phosphorylates serine 2737 located in the PKD-binding site of AKAP-Lbc. Free PKD1 can then phosphorylate HDAC5 an inhibitor of the prohypertrophic transcription factor MEF2. This facilitates its HDAC5 nuclear export, derepression of MEF2 and activation of hypertrophic gene transcription (Figure 1) [59]. 

Subsequent in vivo studies showed that gene-trap mice expressing a PKD1 binding deficient mutant of AKAP-Lbc were not able to sustain compensatory cardiac hypertrophy in response TAC or chronic treatment with hypertrophic agonists [28]. The impaired adaptive response to stress was associated with exacerbated cardiomyocyte apoptosis, early-dilated cardiomyopathy and heart failure. Interestingly, increased apoptosis was linked to a marked transcriptional downregulation of antiapoptotic genes such as Bcl2 and the upregulation of the mRNA encoding pro-apoptotic proteins such as Bax, Gzmm, and Dnm1l (Figure 1) [71]. Therefore, AKAP-Lbc-anchored PKD1 facilitates activation of hypertrophic and cytoprotective gene programs to ensure cardiomyocyte survival and adaptation during the early phase of cardiac remodeling.

### 3.2. AKAP-Lbc Mediates Protection against Doxorubicin-Induced Cardiomyocyte Toxicity

Doxorubicin (Dox) is an anthracycline antibiotic used for the past four decades as an anticancer agent to treat a variety of tumors including leukemia and breast cancer. It exerts its antineoplastic activity by impairing DNA replication, mainly through the inhibition of topoisomerase II, and by promoting the formation of reactive oxygen species (ROS). However, this drug displays severe cardiac side effects, which limit its clinical application and have become a serious concern for cancer survivors [72,73]. Doxorubicin-induced chronic cardiotoxicity is dose-dependent and usually occurs within the first year after treatment. The incidence is about 4% for a doxorubicin dose of 500–550 mg/m^2^, 18% for a dose of 551–600 mg/m^2^ and 36% for a dose exceeding 600 mg/m^2^ [74].

Cardiotoxicity is associated with the ability of Dox to alter Ca^2+^ homeostasis, to affect the expression of sarcomeric proteins, to inhibit the electron transport chain and energy production, and to promote the formation of ROS both in the mitochondria and in the cytoplasm of cardiomyocytes through a series of redox reactions that require iron [75].

ROS production enhances oxidation of DNA [76], proteins and lipids [77], thus causing mitochondrial damage and the activation of cardiomyocyte apoptosis. These effects are reinforced by the profound inhibitory action of Dox on the expression of cytoprotective signaling proteins such the kinase Akt1 and antiapoptotic regulators such as Bcl2 and BclxL [78,79,80]. In the clinic, the only currently available drug that can partially diminish these cardiotoxic effects is dexrazoxane, an iron chelator that reduces Dox-induced ROS formation [81]. However, the fact that a significant number of patients receiving Dox still develop severe cardiac morbidity underscores the urgency of new therapeutical strategies. In this respect, recent research efforts are now focused on identifying cardioprotective signaling pathways that could efficiently reduce cardiac side effects [82]. 

**Figure 2:**
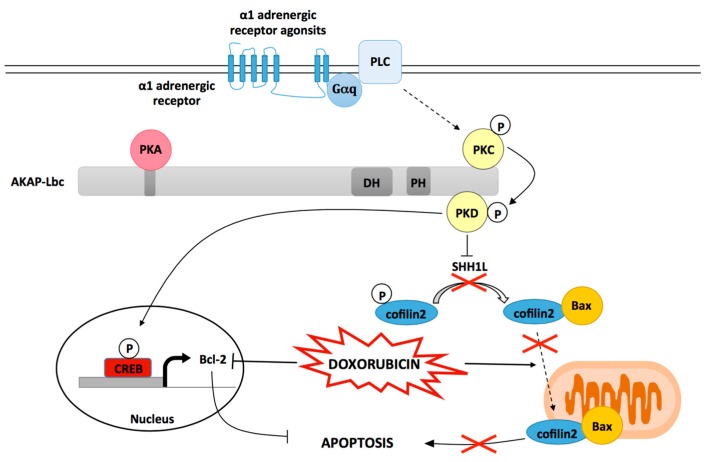
The role of AKAP-Lbc in mediating protection against Dox induced cardiomyocyte toxicity. Scaffolding of PKD by AKAP-Lbc facilitates α1-AR-mediated PKD1 activation resulting in the phosphorylation and inactivation of the phosphatase SSH1L. As a consequence, phosphorylated cofilin2 accumulates and remains sequestrated in the cytoplasm. This inhibits Dox-induced translocation of cofilin2/Bax complexes to mitochondria, and subsequent mitochondrial dysfunction and apoptosis. Activated PKD1 also favors cAMP regulatory element binding protein (CREB)-mediated transcriptional activation of the antiapoptotic gene Bcl-2 otherwise down regulated by Dox treatment.

Several evidences suggest that the activation of α1-ARs significantly reduces the toxic effects that Dox exerts on cardiomyocytes [83]. Indeed, phenylephrine (PE) and dabuzalgron, two α1-AR agonists, confer significant protection against Dox-induced cardiomyocyte apoptosis, pathological cardiac remodeling, and depressed heart function in mice [80,84]. Interestingly, recent studies performed on rat NVMs indicate that these protective effects could be mediated in part by AKAP-Lbc [29]. In particular, it has been shown that short-hairpin RNA (shRNA)-mediated suppression of AKAP-Lbc expression in ventricular myocytes strongly impairs the ability of the α1-AR agonist phenylephrine (PE) to reduce Dox-induced cardiomyocyte apoptosis. AKAP-Lbc-mediated cardiomyocyte protection requires the recruitment of PKD1 and the activation of two PKD1-dependent prosurvival signaling cascades (Figure 2) [29]. 

In the first pathway, the AKAP-Lbc-anchored pool of PKD1 mediates the phosphorylation and activation of the transcription factor cAMP regulatory element binding protein (CREB), which, in turn, promotes upregulation of the antiapoptotic gene Bcl2. This efficiently prevents Dox-induced Bcl2 transcriptional downregulation (Figure 2). In the second pathway, AKAP-Lbc-facilitated activation of PKD1 leads to the phosphorylation and deactivation of the cofilin2-phosphatase slingshot-1L (SSH1L), which increases cofilin2 phosphorylation. This blocks Dox-induced translocation of cofilin2 and Bax complexes to mitochondria, which reduces mitochondrial dysfunction, cytochrome C release, caspase 3 activation and apoptosis (Figure 2) [29,85]. 

Knowing that PKD1 also favors protection against hypoxia and oxidative stress [85], and adaptation against pressure overload-induced early cardiac remodeling [28], one could suggest that this kinase might confer cardiomyocyte protection against a variety of stresses.

It has been shown that infusion of α1-AR agonists such as PE in mice induce a significant upregulation of cardiac AKAP-Lbc expression [57]. This raises the possibility that AKAP-Lbc-mediated cardioprotective signaling could be enhanced by α1-AR agonists in vivo. Based on this assumption, future studies will need to determine the impact of cardiac AKAP-Lbc suppression and overexpression on Dox-induced chronic cardiac side effects. 

### 3.3. The Cardioprotective Role of the AKAP-Lbc/HSP20 Complex

**Figure 3:**
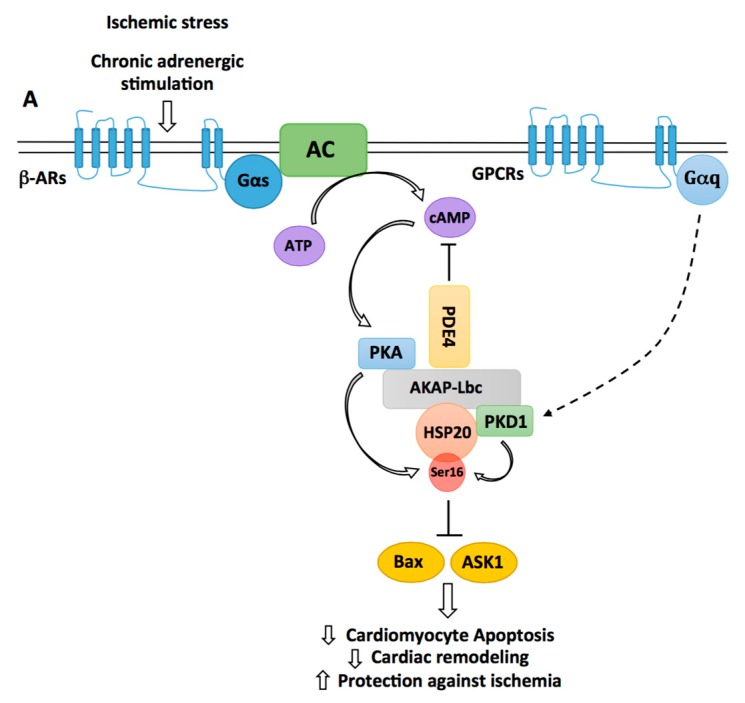
Regulation of HSP20-mediated cardiomyocyte protection by AKAP-Lbc. By recruiting phosphodiesterases 4 (PDE4), AKAP-Lbc maintains a low local concentration of cAMP, which prevents activation of anchored PKA. Chronic β-adrenergic stimulation induces a sustained production of cAMP, which saturates PDE4 and promotes anchored PKA activation. Activated PKA phosphorylates AKAP-Lbc-bound HSP20 on serine 16, an event that has been shown to enhance the cardioprotective function of HSP20. Indeed, phosphorylated HSP20 has been shown to suppress Ask1-dependent signaling and to inhibit Bax leading to reduced cardiomyocyte apoptosis, decreased pathological cardiac remodeling, and increased protection against ischemia. PKD1 can form a complex with HSP20 and promote its phosphorylation on serine 16. The relative contribution of PKA vs. PKD1 to the phosphorylation of HSP20 in vivo remains to be elucidated.

The small heat shock protein HSP20 has been shown to confer sustained protection against cardiac stresses and insults including chronic β-adrenergic stimulation, ischemia/reperfusion (I/R) and Dox exposure. Indeed, transgenic mice with cardiomyocyte-specific overexpression of HSP20 are protected against apoptosis induced by chronic ISO or Dox infusion and develop significantly smaller infarcts when subjected to I/R [47,48,86,87]. HSP20 mediates its antiapoptotic effects through the inhibition of apoptosis signal-regulating kinase 1 (Ask1) and Bax (Figure 3) and the preservation of the pro-survival activity of Akt1 [48,87]. Interestingly, these cardioprotective effects were shown to require phosphorylation of HSP20 on serine 16 by PKA [88]. This was suggested by studies showing that overexpression of a constitutively phosphorylated mutant (S16D) of HSP20 protects adult cardiomyocytes from apoptosis induced by β-adrenergic agonists [47]. In a screening for polymorphisms associated with human dilated cardiomyopathy, it was later found that a single base change of C to T at nucleotide 59 in the N-terminus of HSP20, resulting in an amino acid substitution from proline 20 to leucine (P20L), strongly impaired PKA-mediated phosphorylation of HSP20 [89].

Accordingly, in vitro experiments confirmed that HSP20 P20L was unable to confer protection against I/R-induced cardiomyocyte apoptosis [90]. Recent studies indicate that AKAP-Lbc facilitates PKA-mediated phosphorylation of HSP20 (Figure 3). In particular, it has been shown that AKAP-Lbc stably interacts with HSP20, thus providing a physical link between PKA and the HSP [89]. Importantly, knockdown of AKAP-Lbc and overexpression of a PKA-binding deficient mutant of the anchoring protein in rat NVMs reduce the phosphorylation of HSP20 on serine 16 and increase isoproterenol-induced cardiomyocyte apoptosis [88]. This suggests that phosphorylation of HSP20 by AKAP-Lbc-anchored PKA mediates cardiomyocyte protection. However, it remains to be established whether the anchoring protein favors cardioprotective phosphorylation of HSP20 also in vivo. To this end, future experiments might investigate whether the knockout of AKAP-Lbc in adult hearts affects phospho-HSP20-dependent protective signaling. 

The phosphorylation status of HSP20 is also regulated by PDE4 family members, which directly interact with the heat shock protein (Figure 3) [91]. Recruitment of PDE4 maintains the local concentration of cAMP low, which reduces PKA activation and HSP20 phosphorylation under basal conditions. Upon chronic β-adrenergic stimulation, cAMP levels rise in cardiomyocytes and overcome the hydrolyzing capacity of the PDE, what favors HSP20 phosphorylation [91]. Knowing that PDE4 also interacts with AKAP-Lbc [92], one might raise the hypothesis that AKAP-Lbc might serve as a molecular organizer coordinating the activity of PKA and PDE4 to confer spatiotemporal regulation of HSP20 phosphorylation and antiapoptotic function. 

It has been shown that serine 16 of HSP20 is also a substrate for PKD1 phosphorylation [93]. This suggests that PKD1 could mediate part of its cardioprotective effects through the regulation of HSP20. The kinase has been shown to directly associate with HSP20 [93] but one could assume that AKAP-Lbc could also target PKD1 in proximity of HSP20 [70]. Based on these new findings, it would be interesting to evaluate the relative importance of PKA versus PKD1 as HSP20 kinases in vivo and to determine their impact on the cardioprotective function of HSP20. 

## 4. Conclusions and Perspectives

The ability of AKAPs to integrate and process multiple signals allows them to regulate several physiological and pathological cardiac functions including contraction, heart rhythm, adaptation to stress and transition to heart failure [10,23,24]. In this context, AKAP-Lbc has the peculiarity of coordinating signaling pathways regulating the heart response to hemodynamic or chemical stresses. 

While a number of studies have highlighted the protective role of AKAP-Lbc during the compensated hypertrophic growth of the heart induced by pressure overload and neurohumoral stress, it is currently not known whether this anchoring protein is also involved in later phases of cardiac remodeling. On the one hand, one could speculate that AKAP-Lbc-mediated activation of PKD1, p38α, and mTOR for periods of time that extend beyond the initial phase of compensation might promote deleterious effects through the sustained induction of the fetal gene program and alteration of cardiac contractility [59,94]. On the other hand, however, recent studies indicate that chronic PKD1 and mTOR activation might actually promote cardioprotective effects through the induction of antiapoptotic gene programs [85,95]. To address these contrasting hypotheses future studies using inducible cardiomyocyte-specific AKAP-Lbc knockout mice will need to address the impact of suppressing AKAP-Lbc expression at the end of the compensatory phase on subsequent pathological remodeling.

By facilitating the activation of PKD1 in cardiomyocytes, AKAP-Lbc inhibits cardiomyocyte apoptosis and protects mitochondrial function in response to abrupt increases in the left ventricular afterload and anthracycline (doxorubicin) exposure [28,29]. These antiapoptotic effects are mediated by the upregulation of Bcl2, the inhibition of the translocation of cofilin2 and Bax to mitochondria, and possibly HSP20. Therefore, strategies aimed at stimulating the activity of AKAP-Lbc-anchored PKD1 might represent a possible way to prevent early cardiac dysfunction in the stressed heart. Knowing that α1-ARs are upstream activators of the AKAP-Lbc/PKD1 signaling pathway, one could propose the use of α1-ARs selective agonists as cardioprotective agents. In this context, dabuzalgron, an oral α1A-AR agonist that was originally developed to treat urinary incontinence, could be repurposed to reduce the cardiac side effects of Dox-based anticancer chemotherapy and possibly to limit cardiomyocyte apoptosis in hemodynamically challenged hearts [84]. 

We recently identified a small molecule able to inhibit AKAP-Lbc-mediated RhoA activation and oncogenic signaling in metastatic prostate cancer cells [96]. While these studies suggest that AKAP-Lbc might represent a potential target in anticancer therapy, one has to consider that compounds inhibiting AKAP-Lbc signaling could potentially interfere with the protective function of the anchoring protein in cardiac cells. Based on this possibility, it will be crucial to carefully evaluate the chronic effect of such molecules on cardiac function.

In conclusion, based on the experimental evidence accumulated over the past decade one could postulate that manipulating the activity of cardioprotective signaling enzymes anchored to AKAP-Lbc might confer early cardiac protection. However, additional investigations will be necessary to decipher the impact of interfering with the AKAP-Lbc signaling properties on late cardiac remodeling and transition to heart failure.
